# Weight gain promotes the progression of IgA nephropathy in Asians: A protocol for systematic review and meta-analysis

**DOI:** 10.1097/MD.0000000000031824

**Published:** 2022-11-18

**Authors:** Meixi Liu, Chunguang Yi, Tianying Chang, Di Zou, Shoulin Zhang

**Affiliations:** a Affiliated Hospital of Changchun University of Chinese Medicine, Changchun, Jilin Province, China; b Evidence-based Office, Affiliated Hospital of Changchun University of Chinese Medicine, Changchun, Jilin Province, China; c Nephropathy Department, Affiliated Hospital of Changchun University of Chinese Medicine, Changchun, Jilin Province, China.

**Keywords:** Asian, BMI, IgAN, weight gain

## Abstract

The relationship between weight gain and progression of IgA nephropathy (IgAN) has been investigated by many researchers but remains controversial. The incidence of IgAN is significantly higher in Asia than in other regions. Therefore, we investigated the relationship between weight gain and primary IgAN in the Asian population.

**Methods and analysis:**

Seven databases were retrieved up to now. We stratified the included population by body mass index (BMI) and performed a meta-analysis of associated risk factors.

**Objectives:**

In this study, Asian IgAN patients with different BMI were grouped together to clarify the relationship between BMI and IgAN progression in Asian populations, so as to provide more ideas and treatment means for the prevention and treatment of IgAN in the future.

## 1. Introduction

The relationship between obesity and diseases of various systems is a hot issue worldwide, and most diseases have been confirmed linked to obesity.^[1]^ IgAN is a common clinical primary renal disease characterized by the deposition of IgA in the mesangial region of the glomerulus.^[2,3]^ About 10% to 20% of patients will progress to end-stage renal disease (ESRD) within 10 years, so it is necessary to explore the factors associated with the progression of IgAN to prolong the survival time of patients and improve the quality of life of patients. In recent years, there have been different views on the impact of BMI on IgAN. Some authors believe that the progression of the IgAN is related to excessive BMI, and others agree that the progression of IgAN is associated with being underweight. In addition, the geographical distribution of IgAN also showed obvious differences.^[4]^ The incidence is high in Asia (40%–50%), moderate in Europe (20%–30%), and the lowest in Africa (<5%). Based on the high incidence of IgAN in Asia, we conducted the first systematic review and meta-evaluation of the association between BMI and IgAN progression in Asian populations to clarify the relationship between BMI and IgAN progression in Asian populations.

The classification standards for BMI are not the same, so we need to combine different BMI classifications before conducting a meta-analysis to help us analyze the correlation between different BMI levels and the progression of IgAN. Due to the differences in sample size and research methods, the results of individual studies may be inaccurate. Therefore, during the process of meta-analysis, we will standardize the results of the studies, and if necessary, we will also conduct a subgroup analysis to find out the factors that influenced the results of the article.

## 2. Methods

This review will follow the Preferred Reporting Project for Systematic Review and Meta-Analysis (PRISMA) guidelines, see S1 file, Supplemental Digital Content, http://links.lww.com/MD/H982. This review was registered in the International Prospective Register of Systematic Reviews (PROSPERO) with the identifier CRD42021253292.

### 2.1. Search strategy

We will search in Pubmed for MESH and free words for standard medicine first. We will use the following terms as search terms to conduct MESH and free word searches, respectively: Glomerulonephritides IGA, Berger’s Disease, Bergers Disease, IGA Glomerulonephritis, Nephropathy IGA, IgAN 1, Nephropathy 1 Iga, Immunoglobulin A Nephropathy, Nephropathy, Immunoglobulin A, Nephritis, IGA Type, IGA Type Nephritis, Berger Disease, IgAN, BMI, Index Body Mass, Quetelet Index, Index Quetelet, Quetelet’s Index, Quetelets Index, Body Weights, Weight Body, and Weights Body. To prevent the omission of literature that may be closely related to the study, we will review references cited in all studies selected for review, and we will select literature in English and Chinese for inclusion in the study. Two reviewers (MXL and DZ) will search papers published from the inception to now independently. We will search the following 7 electronic databases, including PubMed/MEDLINE, EMBASE, Cochrane Library, Chinese Science and Technology Journals (CNKI, Sinomed, VIP, and Wan Fang). If 1 of the reviewers felt that the article met the inclusion criteria it will be included. In the event of disagreement on literature, a third reviewer (TYC) will decide whether the literature will be included or not. In the month of literature completion, PubMed/MEDLINE will be searched to capture all articles published after the original search date.

### 2.2. Study selection

We will include articles on the relationship between BMI and IgAN; in addition, the research needs to meet:

(1) case–control, cohort, and cross-sectional studies published in English and Chinese,
(2) the included studies need to be classified into at least 3 grades according to BMI classification,
(3) biopsy confirmed primary IgAN,
(4) the research object is Asian population.

The exclusion criteria are as follows:

(1) case analysis and review articles,
(2) articles not grouped according to BMI classification or grouped into <3 groups,
(3) patients with secondary IgAN were excluded,
(4) articles that are grouped by other factors besides BMI, lack original data and duplicate data,
(5) the subjects of the study are people outside Asia.

**Figure 1.**
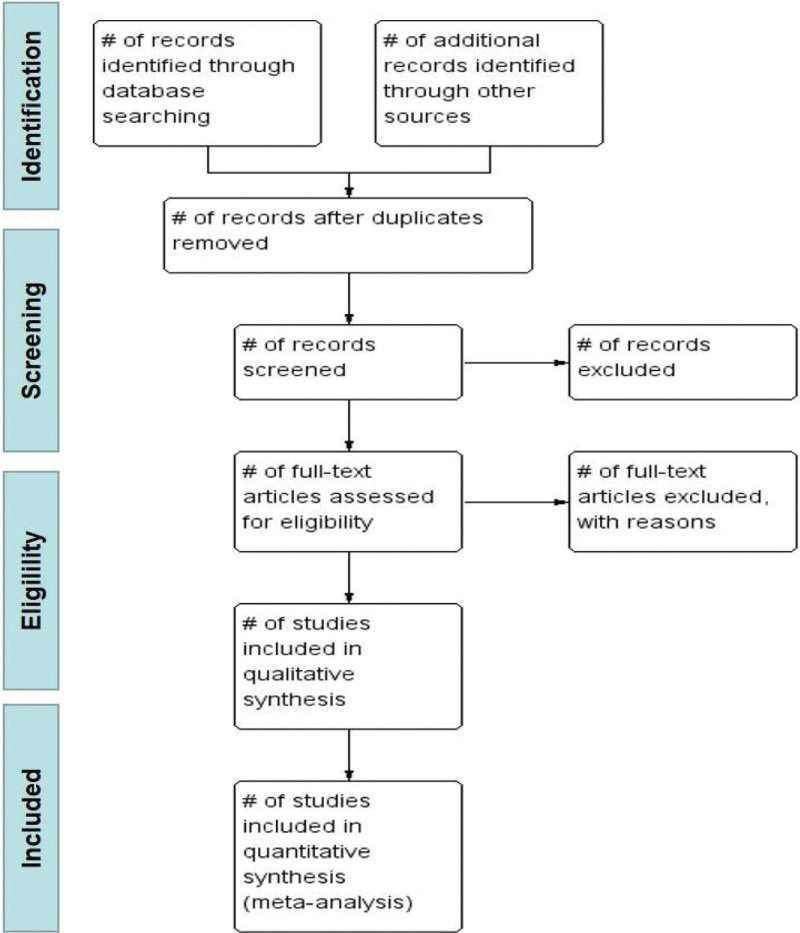
PRISMA flow diagram.

The selecting process will follow the PRISMA flow diagram, see Figure 1.

### 2.3. Quality assessment

Since 7 of the included studies were cohort studies and 1 was a cross-sectional study, so we will use the Newcastle-Ottawa-Scale (NOS)^[5]^ score to evaluate the quality of the cohort studies. Furthermore, the American Agency for Health Care Quality and Research (AHQR)-recommended method was used to evaluate the literature quality of cross-sectional studies.^[6]^ For cross-sectional studies, if the answer is “no” or “unclear,” the item will be rated “0”; if the answer is “yes,” the item will be rated “1.”

For all studies, we will use “☆”, “★” and “★★” to judge the overall quality of the included studies, which respectively represent 0, 1, and 2 scores. A study was scored when both raters (MXL and DZ) agreed that the individual scores were consistent. If the results of the 2 reviewers differ significantly, the 2 reviewers’ scores differ over 2 points, and a third reviewer (TYC) will conduct the study to arrive at the final opinion. Literature quality was assessed as follows: cohort studies: low quality <5, moderate quality 5–6, high quality >7; cross-sectional studies: low quality = 0–3; moderate quality = 4–7; high quality = 8–11. The quality evaluation process of the literature is presented in the included literature quality evaluation table, see Table 1.

**Table 1:** Included literature quality evaluation table.

Study	Selection							Comparability	Exposure					Scores
Cohort study	Adequate definition of cases	Representative liveness of the cases		Selection of controls		Definition of controls		Control for important factor	Ascertainment of exposure		Same method of ascertainment for cases and controls	Non-response rate		Scores
Cross-sectional study	Source of information	Exposed or unexposed	Time period		Continuity of patients		Hide the results	Outcome index detection	Explain the patient Exclusions	Control for confounding factors	Handling of missing values	Response rate and completeness	Follow-up result	Scores

* Newcastle-Ottawa-Scale (NOS) was used in the first cohort studies, and the American Agency for Health Care Quality and Research (AHQR) was used in the cross-sectional study.

☆ = 0, ★ = 1, ★★ = 2.

### 2.4. Data extraction

We will extract relevant trial design data, including publication year, nationality, research type, sample size, gender, age, and outcomes as following, including 24-hour urine protein, TC, CREA, UA, eGFR, and incidence of hypertension. All the collection results were filled into the table for basic clinical indicators of the included study population and the table for basic characteristics of the included studies, see Tables 2 and 3.

**Table 2:** Basic clinical indicators of the included study population.

Study	Hypertension (%)	TC (mg/dL)	UA (umol/L)	CREA (mg/dL)	24h urinary protein (g/24h)	eGFR (mL/min/1.73m^2^)
						

NA = no data.

**Table 3:** Basic characteristics of the included studies.

Serial number	Study	Year	Country	Study type	Sample size	Man/woman	Age
							

NA = no data.

### 2.5. Assessment of BMI

At present, according to the WHO BMI cutoff point,^[7]^ the BMI classification is mainly divided into underweight (<18.5 kg/m^2^), normal (18.5–24.9 kg/m^2^), overweight (25–29.9 kg/m^2^), and obesity (30 kg/m^2^). Therefore, we will make a bar chart of the BMI distribution of the literature and divide the literature into different groups according to the similarity of the BMI classification method. They are as follows: the relative normal-weight group, the overweight/obese group, and the low-weight group.

### 2.6. Assessing risk of bias

Two researchers (MXL and DZ) will assess the risk of bias for included articles independently according to the Cochrane risk of bias (ROB) tool for interventions including:

(1) sequence generation,
(2) allocation concealment,
(3) blinding of participants and personnel,
(4) blinding of outcome assessment,
(5) incomplete outcome data,
(6) selective reporting, and
(7) other bias.

The assessment will be graphed and use Review Manager 5.3 software.

### 2.7. Data synthesis

Odds ratios (ORs) and 95% confidence intervals analyzed dichotomous variables. The fixed-effect model was the prior model for the continuous data effect model. We will use the *I*^2^ statistic and *χ*^2^ test to evaluate the heterogeneity between studies. If the heterogeneity results showed *P* < .10, there was heterogeneity between the 2 groups. *I*^2^ will be used to assess the heterogeneity of the included studies. The fixed-effects model will be chosen when the heterogeneity is <50%, and the random-effects model will be used if the heterogeneity is >50%. If the number of articles is >10, publication offsets will be assessed.

We will perform a meta-analysis of 6 outcomes of the relative normal-weight group, overweight/obesity group and the low-weight group and obtain the forest plot. Sensitivity analysis for each data will be conducted to evaluate the results’ robustness. If we find the direction of heterogeneity of the article has changed significantly after eliminating an article, the literature will be regarded as the source of heterogeneity. The reasons for this result will be further analyzed.

## 3. Discussion

Obesity has been linked to many diseases.^[8,9]^ Many studies have shown that obesity and increased central body fat are related to diabetes, hypertension, cardiovascular and cerebrovascular diseases, sleep apnea, and many chronic diseases that seriously affect physical and mental health. Loss of weight slightly was found to improve these comorbidities in the study.^[10]^ Therefore, if we find that obesity is related to a certain disease, we can help treat the disease or delay the further development of the disease by weight control. BMI (kg/m^2^) is the main criterion for evaluating obesity. The current classification of BMI is mainly based on the standards established by WHO.^[11,12]^ BMI < 18.5 kg/m^2^ is underweight, 25–29.9 kg/m^2^ is overweight, BMI > 30 kg/m^2^ is obese, BMI > 40 kg/m^2^ (or ≥ 35kg/m^2^ with comorbidities) is severely obese. There are significant regional differences in IgAN, so racial differences are an issue that we cannot ignore. Ethnic factors are related to ethnicity or large populations grouped according to common characteristics and customs, divided into intrinsic and extrinsic factors. Extrinsic ethnic factors include culture and environment, while intrinsic ethnic factors help define and identify subpopulations, including genetics, body composition, and other factors, therefore, whether obesity is associated with IgAN progression in Asian populations. Due to ethnic differences in various continents, WHO has also formulated different obesity definition points, including Asia. In addition to WHO, many regions and countries have developed specific BMI standards. In recent years, people have also begun to explore the relationship between obesity and IgAN. However, we found some disagreement about the relationship between BMI and IgAN. Some researchers believe that an increase in BMI is closely related to the progression of IgAN,^[13]^ while others believe that a low BMI is related to the progression of IgAN.^[14]^ Based on that, we compared the low-weight group with the normal-weight group.

In conclusion, through this article, we hope to verify the relationship between BMI and IgAN, and provide more evidence to support for the treatment and prognosis of IgAN, so as to delay the progression of IgAN and improve the quality of survival of patients.

## Author contributions

MXL searched and selected literatures, and wrote the article. TYC and DZ searched and selected literatures, CGY controlled the quality of articles, and guided the technology. SLZ reviewed topic selection, feedback articles, and guided the technology.

**Conceptualization:** Meixi Liu.

**Data curation**: Meixi Liu, Chunguang Yi, Di Zou.

**Formal analysis**: Meixi Liu, Tianying Chang, Di Zou.

**Funding acquisition**: Shoulin Zhang.

**Investigation**: Shoulin Zhang.

**Methodology**: Tianying Chang, Di Zou.

**Project administration**: Shoulin Zhang.

**Resources**: Meixi Liu, Chunguang Yi, Tianying Chang, Di Zou.

**Software**: Meixi Liu, Chunguang Yi, Tianying Chang.

**Supervision**: Chunguang Yi, Shoulin Zhang.

**Validation**: Shoulin Zhang.

**Visualization**: Meixi Liu, Tianying Chang, Shoulin Zhang.

**Writing – original draft:** Meixi Liu.

**Writing – review & editing**: Meixi Liu, Chunguang Yi, Tianying Chang, Shoulin Zhang.

## Supplementary Material
